# Inhibition of the TGFβ signalling pathway by cGMP and cGMP‐dependent kinase I in renal fibrosis

**DOI:** 10.1002/2211-5463.12202

**Published:** 2017-03-01

**Authors:** Elisabeth Schinner, Veronika Wetzl, Andrea Schramm, Frieder Kees, Peter Sandner, Johannes‐Peter Stasch, Franz Hofmann, Jens Schlossmann

**Affiliations:** ^1^ Department of Pharmacology and ToxicologyUniversity of RegensburgGermany; ^2^ Novartis Pharma GmbHNurembergGermany; ^3^ Bayer Pharma AGWuppertalGermany; ^4^ Institute of Pharmacology and ToxicologyTechnical University of MunichGermany

**Keywords:** cGMP‐dependent protein kinase I, cyclic guanosine monophosphate, renal fibrosis, soluble guanylate cyclase stimulation

## Abstract

Agents that enhance production of nitric oxide (NO) and cyclic guanosine monophosphate (cGMP) ameliorate the progression of renal fibrosis. However, the molecular mechanism of this process is not fully understood. We hypothesize that the antifibrotic effects of cGMP and cGMP‐dependent kinase I (cGKI) are mediated via regulation of the TGFβ signalling pathway, both via ERK and the Smad‐dependent route. Kidney fibrosis was induced by unilateral ureter obstruction (UUO) in wild‐type and cGKI‐deficient (cGKI‐KO) mice. The cGMP/cGKI signalling pathway was activated by application of the soluble guanylate cyclase (sGC) stimulator BAY 41‐8543 (BAY), beginning 1 day after UUO. After 7 days, the antifibrotic effects of BAY were analysed by measuring mRNA and protein expression of characteristic fibrotic biomarkers. The effects of cGMP/TGFβ on cultured fibroblasts were also analysed *in vitro*. BAY application influenced the activity of the extracellular matrix (ECM)‐degrading matrix metalloproteases (MMP2 and MMP9) and their inhibitor tissue inhibitors of metalloproteinase‐1, the secretion of cytokines (e.g. IL‐6) and the expression pattern of ECM proteins (e.g. collagen, fibronectin) and profibrotic mediators (e.g. connective tissue growth factors and plasminogen‐activator inhibitor‐1). Activation of the cGMP/cGKI signalling pathway showed protective effects against fibrosis which were mediated by inhibition of P‐Erk1/2 and translocation of P‐smad3. The elucidation of these signalling mechanisms might support the development of new therapeutic options regarding cGMP/cGKI‐mediated antifibrotic actions.

**Abbreviations**
cGKI: cGMP‐dependent protein kinase I
cGKI‐KO: cGKI‐knockout
cGMP: cyclic guanosine monophosphate
Co‐IP: coimmunoprecipitation
Col1a1: collagen1a1
CTGF: connective tissue growth factor
ECM: extracellular matrix
ERK1/2: extracellular‐signal regulated kinase
GTP: guanosine triphosphate
MMPs: matrix metalloproteinases
NO: nitric oxide
PAI‐1: plasminogen‐activator inhibitor‐1
sGC: soluble guanylyl cyclase
TGFβ: transforming growth factor β
TIMP: tissue inhibitors of metalloproteinases
UUO: unilateral ureter obstruction
wt: wild‐type
αSMA: α‐smooth muscle actin

Fibrosis is characterized by excessive expression of extracellular matrix (ECM). Fibrogenic factors promote the fibrotic process such as transforming growth factors (TGFβ), plasminogen‐activator inhibitor‐1 (PAI‐1) or connective tissue growth factors (CTGF) 1. TGFβ is involved in the differentiation of fibroblasts to myofibroblasts, which are characterized by the expression of α‐smooth muscle actin (αSMA). Myofibroblasts synthesize ECM proteins including collagen and fibronectin, and they secrete cytokines, for example, IL‐6.

In addition, synthesis and degradation of ECM proteins are determined by metalloproteinases (MMPs) and tissue inhibitors of metalloproteinases (TIMPs). Their expression pattern is regulated by MAPK/Erk kinase, which promotes the progression of fibrosis 2, 3.

We evaluated the effects of the soluble guanylate cyclase (sGC) stimulator BAY 41‐8543 (BAY) on the fibrotic kidney. Under physiological conditions, sGC can be activated by nitric oxide (NO). Activated sGC synthesizes the second messenger cyclic guanosine monophosphate (cGMP) which then stimulates cGMP‐dependent protein kinases (cGK) 4. We have previously reported that cGMP suppresses renal fibrosis in particular via cGKIα, an isoform of cGK. cGKIα is expressed in fibroblasts and myofibroblasts, which are excessively produced after unilateral ureter obstruction (UUO) 5. Protective effects of sGC stimulation on renal fibrosis in rats have already been shown 6, 7. Thereby, BAY reduced apoptosis and macrophage infiltration after relief of UUO 7, and Sharkovska *et al*. 6 reported that sGC stimulation improved creatinine clearance in hypertensive renin‐transgenic rats. However, the molecular mechanism by which cGMP via cGKI affects the development of kidney fibrosis has not yet been fully elucidated. Therefore, we analysed the impact of BAY on fibrosis in a mouse model of UUO using cGKI‐knockout (cGKI‐KO)‐mice. The present study investigates the functional role of sGC stimulation in the fibrotic process, the signalling pathway as well as the underlying mechanisms involved.

## Results

### Effect of BAY and function of cGKI on the mRNA expression of different fibrotic biomarkers

**Figure 1:**
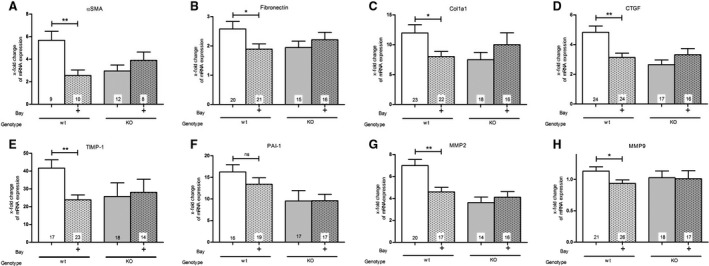
Effect of BAY in wt‐ and cGKI‐KO‐kidneys on the mRNA expression levels of (A) αSMA, (B) fibronectin, (C) Col1a1, (D) CTGF, (E) TIMP‐1, (F) PAI‐1, (G) MMP2 and (H) MMP9. In wt‐mice, BAY caused a significant decrease in the mRNA expression of (A) αSMA, (B) fibronectin, (C) Col1a1, (D) CTGF, (E) TIMP‐1, (G) MMP2 and (H) MMP9 with the exception of (F) PAI‐1. In cGKI‐KO‐mice sGC stimulation showed no significant decrease in the mRNA levels (A–H). The results are shown as the x‐fold change in mRNA expression in the fibrotic kidney relating to the opposite healthy kidneys whose mRNA expression was set to one. In each mouse strain the untreated mice were compared with BAY‐treated mice. Significant differences between two groups are indicated with asterisks (*P < 0.05, **P < 0.01). The columns show the number of animals which were used. The right columns illustrate the data of GKI‐KO‐mice and patterned columns the data of BAY‐treated mice.

As marker for fibrosis induction, we examined the mRNA levels of αSMA, fibronectin, collagen1a1 (Col1a1), CTGF, TIMP‐1, PAI‐1, MMP2 and MMP9 (Fig. 1). One week after UUO surgery, the mRNA levels were elevated in comparison to the contralateral control kidney. Especially, αSMA (Fig. 1A), Col1a1 (Fig. 1C), TIMP‐1 (Fig. 1E), PAI‐1 (Fig. 1F) and MMP2 (Fig. 1G) were strongly increased by UUO. In contrast, the mRNA expression of fibronectin (Fig. 1B) and CTGF (Fig. 1D) were only moderately upregulated, and MMP9 (Fig. 1H) was nearly unchanged.

To examine the role of NO/cGMP signalling in renal fibrosis, we injected the sGC stimulator BAY. A significant raise of cGMP in kidney tissues of BAY‐treated mice in comparison to untreated mice indicating a BAY‐induced stimulation of sGC was measured (Fig. S1). BAY treatment decreased the mRNA expression of all investigated biomarkers of fibrosis with the exception of PAI‐1 (Fig. 1F). The expression of PAI‐1 was reduced but the difference did not reach significance. To explore whether cGKI is involved in the impact on the fibrotic process, we analysed cGKI‐KO‐mice. As previously reported, untreated cGKI‐KO‐mice showed less mRNA expression than untreated wild‐type (wt) mice 5. However, the mRNA expression of cGKI‐KO‐mice was not influenced by BAY application (Fig. 1).

### Effect of BAY and role of cGKI on the protein expression of: αSMA, fibronectin, Col1a1 and total collagen

**Figure 2:**
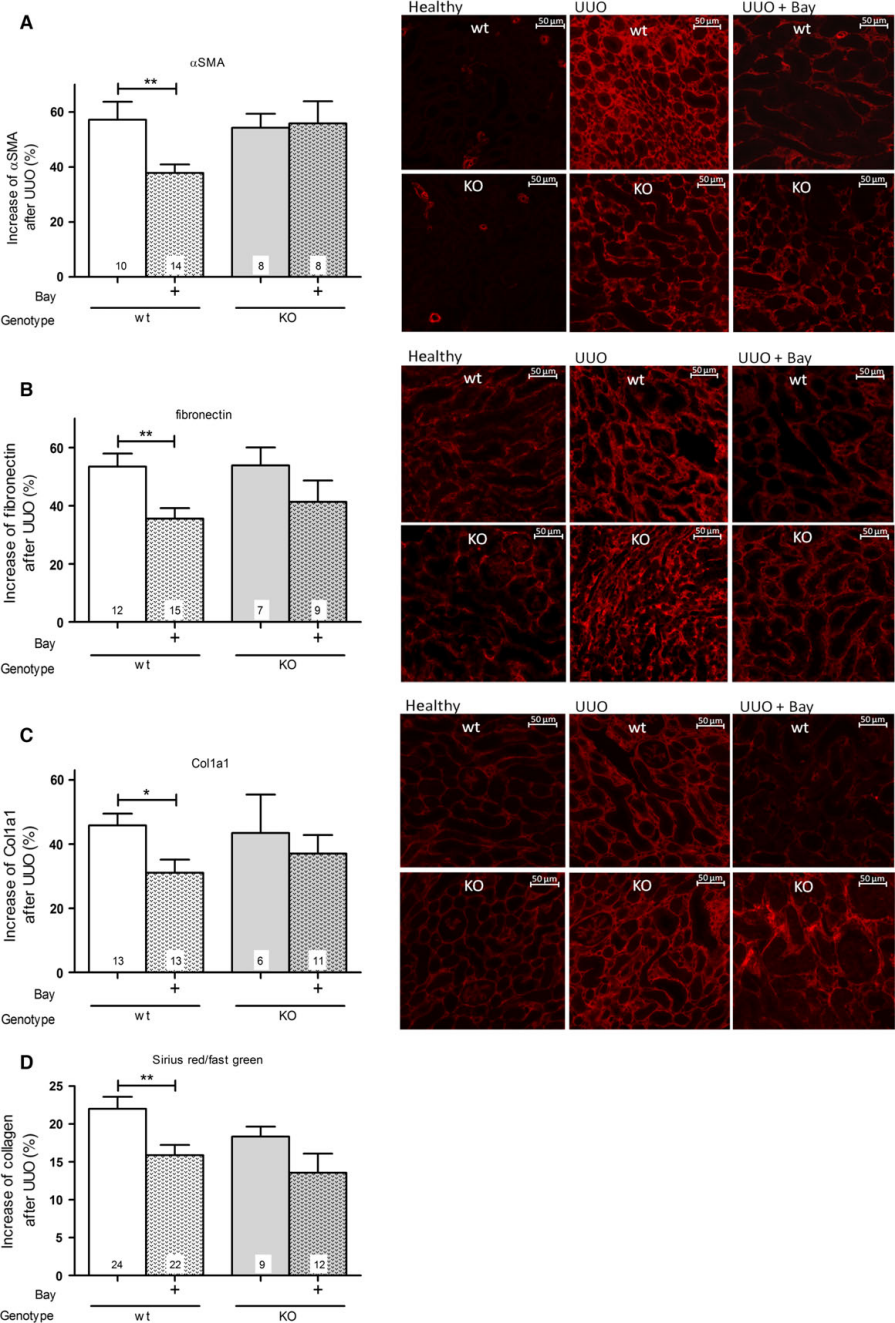
Effect of BAY in wt‐ and cGKI‐KO‐kidneys on the protein levels of (A) αSMA, (B) fibronectin, (C) Col1a1 and (D) total Collagen. Metamorph offline was used for the quantification of fluorescence‐intensity of (A) αSMA, (B) fibronectin and (C) Col1a1. Immunofluorescence staining of (A) αSMA (Alexa488, shown in red), (B) fibronectin (Alexa647, shown in red) and (C) Col1a1 (Alexa647, shown in red) in healthy, UUO‐untreated and UUO‐BAY‐treated kidneys of wt‐ and cGKI‐KO‐mice. Total collagen levels in the kidneys were measured by Sirius red/fast green staining (D). The protein expression of (A) αSMA, (B) fibronectin, (C) Col1a1 and (D) total Collagen was significantly diminished in wt‐mice, but not in cGKI‐KO‐mice by BAY. Thereby, the increase in protein by UUO was related to the healthy kidney. In each mouse strain the untreated mice were compared with BAY‐treated mice. Significant differences between two groups are indicated with asterisks (*P < 0.05, **P < 0.01). The columns show the number of animals which were used. The right columns illustrate the data of GKI‐KO‐mice and patterned columns the data of BAY‐treated mice.

After UUO, the interstitial accumulation of protein expression of αSMA, fibronectin and Col1a1 was increased in wt‐ and cGKI‐KO kidneys as demonstrated by immunofluorescence analysis (Fig. 2A–C). The quantitative analysis revealed that BAY significantly reduced the protein expression of αSMA, fibronectin and Col1a1 in wt‐, but not in cGKI‐KO‐kidneys (Fig. 2A–C). The same pattern was present when we used the Sirius red/fast green staining for total collagen. In wt‐, but not in cGKI‐KO‐kidneys, sGC stimulation by BAY significantly downregulated the level of total collagen (Fig. 2D).

### Effect of BAY and function of cGKI on the activity or protein expression of TGFβ target genes

**Figure 3:**
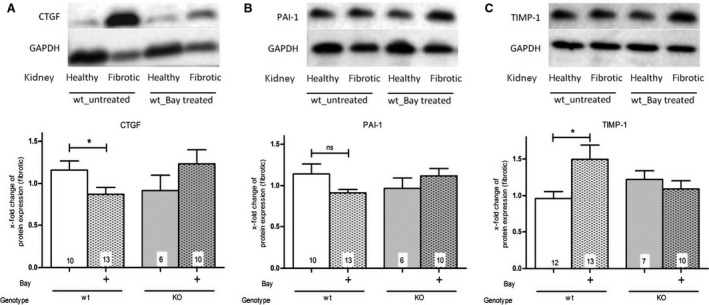
Effect of BAY in wt‐ and cGKI‐KO‐kidneys on the protein levels of (A) CTGF, (B) PAI‐1 and (C) TIMP‐1. The immunoblots show the protein expression of CTGF (A), PAI‐1 (B) and TIMP‐1 (C) in wt‐mice of healthy and fibrotic kidneys (BAY treated or untreated). The graphs statistically compare the protein expression of CTGF (A), PAI‐1 (B) and TIMP‐1 (C) in fibrotic wt‐ and cGKI‐KO‐kidneys. CTGF (A) and TIMP‐1 (C) are significantly influenced by BAY in wt‐, but not in cGKI‐KO‐kidneys. Thereby, each value of the used markers of wt‐ and cGKI‐KO‐kidneys is related to the mean value of untreated fibrotic wt‐kidneys which was set to one and normalized to the corresponding GAPDH. The protein expression of GAPDH was changed by UUO but not by BAY. Therefore, the statistic compares only fibrotic kindeys. Significant differences between two groups are indicated with asterisks (*P < 0.05). The columns show the number of animals which were used. The right columns illustrate the data of GKI‐KO‐mice and patterned columns the data of BAY‐treated mice.

**Figure 4:**
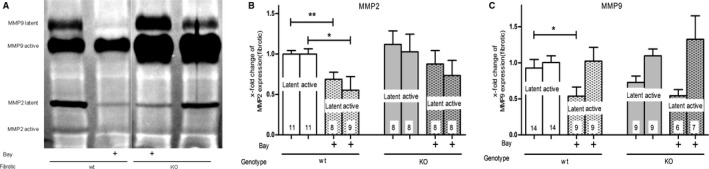
Effect of BAY in fibrotic wt‐ and cGKI‐KO‐kidneys on the activity of MMP2 and MMP9. Latent and active MMP2 and MMP9 of fibrotic wt‐ and cGKI‐KO‐kidneys were determined by Gelatin zymography assays (A). In fibrotic wt‐kidneys, latent and active MMP2 (B) and latent MMP9 (C) were significantly reduced after BAY application. In cGKI‐KO‐kidneys BAY showed no effects regarding the activity of MMP2 and 9. Each value of wt‐ and cGKI‐KO‐kidneys is related to the mean value of untreated fibrotic wt‐kidneys which was set to one. Significant differences between two groups are indicated with asterisks (*P < 0.05, **P < 0.01). The columns show the number of animals which were used. The right columns illustrate the data of GKI‐KO‐mice and patterned columns the data of BAY‐treated mice.

As expected, UUO increased the protein expression of the TGFβ target gene CTGF in comparison to the healthy kidney (Fig. 3A). The quantitative analysis, which compared only fibrotic kidneys, confirmed that the protein expression of CTGF was significantly diminished by BAY in fibrotic wt‐kidneys. However, treatment of cGKI‐KO‐mice did not result in a reduction of CTGF (Fig. 3A). Figure 3B demonstrates that PAI‐1‐expression was not significantly influenced by BAY. The protein expression of TIMP‐1 was not changed by UUO in comparison to the contralateral healthy kidney (Fig. 3C). Intriguingly, TIMP‐1 was significantly higher in BAY treated than in untreated fibrotic wt‐mice. In cGKI‐KO‐mice, we detected no increase in TIMP‐1 expression following BAY administration (Fig. 3C). The latent and active forms of MMP2 and the latent forms of MMP9 were elevated, but the active forms of MMP9 were reduced by UUO (data not shown). In agreement with the increase in TIMP‐1, which is an inhibitor of MMPs, the latent and active forms of MMP2 (Fig. 4A,B) and the latent forms of MMP9 (Fig. 4A,C) were significantly diminished by BAY. This was again only observed in wt‐, but not in cGKI‐KO‐kidneys.

### Effect of cGMP/cGKI on the TGFβ signalling pathway

**Figure 5:**
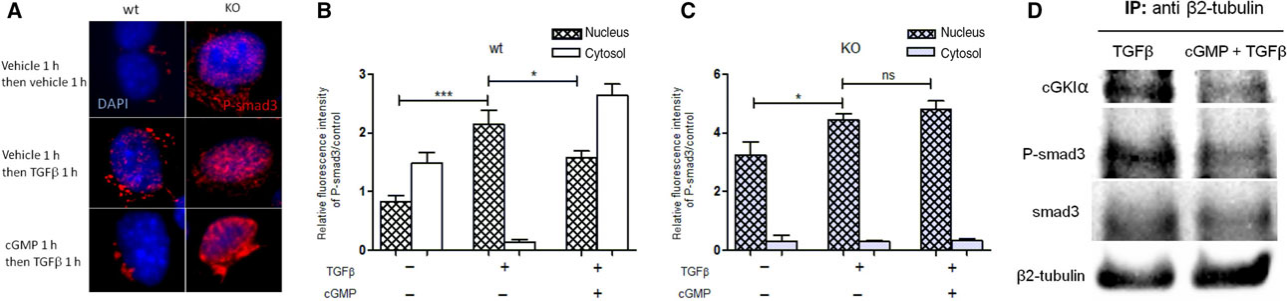
Effect of cGMP on the TGFβ/smad signalling pathway in renal wt‐ and cGKI‐KO‐fibroblasts. Serum‐starved fibroblasts which were isolated from wt‐ (left sided) and cGKI‐KO‐kidneys (right sided) were pretreated with 8Br‐cGMP (1 mm) or vehicle (control) for 1 h, followed by TGFβ (2 ng·mL^−1^) or vehicle for 1 h and stained with P‐smad3 (Alexa647 anti‐rabbit, shown in red) and DAPI (shown in blue) (A). The statistic of the fluorescence intensity of P‐smad3 in nucleus or cytosol is demonstrated in (B) wt‐fibroblasts and (C) cGKI‐KO‐fibroblasts. Wt‐fibroblasts were stimulated with TGFβ or cGMP/TGFβ for the coimmunprecipitation (Co‐IP) analysis which was performed with whole cell extracts using anti‐β2‐tubulin antibody. The blot was probed with anti‐cGKIα and smad3, then after stripping with anti‐P‐smad3 and then after stripping with anti‐β2‐tubulin (D). Significant differences between two groups are indicated with asterisks (*P < 0.05, ***P < 0.001). The experiments were repeated five to seven times.

At first we analysed the influence of cGMP/cGKI on the TGFβ/smad signalling pathway. Isolated fibroblasts of wt‐ (left side of Fig. 5A) and cGKI‐KO‐kidneys (right side of Fig. 5A) were pretreated with cGMP or vehicle followed by exposure to TGFβ or vehicle (Fig. 5A). We quantified the intranuclear and extranuclear fluorescence intensity of P‐smad3 respectively. Figure 5B shows that TGFβ treatment significantly enhanced nuclear fluorescence intensity of P‐smad3 but pretreatment with cGMP significantly limits nuclear translocation of P‐smad3 in fibroblasts of wt‐kidneys in the presence of TGFβ. cGMP alone had no effects (data not shown). Intriguingly in fibroblasts of cGKI‐KO‐kidneys pretreatment with cGMP did not change the translocation of P‐smad3 (Fig. 5C). In contrast to P‐smad3, P‐smad2 was not influenced by preincubation with cGMP (data not shown). Isolated fibroblasts expressed sGC but during culturing the expression of sGC was downregulated (data not shown). Therefore, we stimulated the cells only with cGMP and not with the sGC stimulator BAY. Furthermore, we quantified the total cellular fluorescence intensity of P‐smad3 which was significantly increased by TGFβ treatment but interestingly not significantly changed by cGMP pretreatment (Fig. S2). In pulmonary artery smooth muscle cells activation of cGMP/PKG limited TGFβ‐induced nuclear translocation of smad3 by sequestering smad3 with cytosolic β2‐tubulin 8. Therefore, we performed a coimmunoprecipitation (Co‐IP) of stimulated fibroblasts to check whether smad3 and β2‐tubulin form a cGMP‐dependent complex. Figure 5D shows that β2‐tubulin antibody precipitated cGKIα, P‐smad3 and smad3 in TGFβ‐ and cGMP/TGFβ‐stimulated fibroblasts. However, in contrast to Gong *et al*. 8, there was no increase in the intensity of the bands after pretreatment with cGMP.

**Figure 6:**
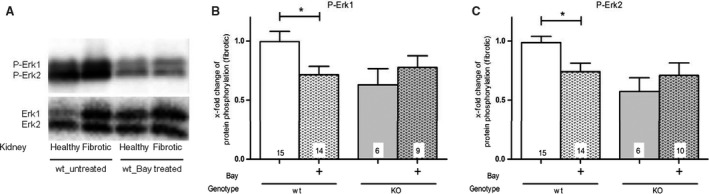
Effect of BAY on the TGFβ/Erk signalling pathway in wt‐ and cGKI‐KO‐mice (A) Representative western blots of Erk1/2 in healthy/fibrotic kidney tissue of wt‐mice untreated/treated with BAY. The graphs statistically compare the protein expression of P‐Erk1 (B) and P‐Erk2 (C) in fibrotic wt‐ and cGKI‐KO‐kidneys. P‐Erk1 (B) and P‐Erk2 (C) were significantly reduced by BAY in wt‐, but not in cGKI‐KO‐kidneys. Thereby, each value of P‐Erk1/2 of wt‐ and cGKI‐KO‐kidneys is related to the mean value of untreated fibrotic wt‐kidneys which was set to one and normalized to the corresponding Erk1/2. Significant differences between two groups are indicated with asterisks (*P < 0.05). The columns show the number of animals which were used. The right columns illustrate the data of GKI‐KO mice and patterned columns the data of BAY‐treated mice.

Second, the phosphorylation of Erk1 and Erk2 (P‐Erk1/2) was assessed. UUO increased the phosphorylation and the protein expression of Erk1 and Erk2 (Fig. 6A). Immunoblots with antibodies against total Erk1 and Erk2 demonstrated that their expression is increased by UUO, but not changed by Bay administration (Fig. 6A). Accordingly, in Fig. 6B,C only fibrotic kidneys are compared and P‐Erk1/2 is normalized to Erk1/2 and related to untreated fibrotic wt‐kidneys. sGC stimulation caused a significant decrease in P‐Erk1 and P‐Erk2 in fibrotic kidneys of treated in contrast to untreated wt‐mice. This BAY‐induced decrease of Erk1/2 phosphorylation in fibrotic wt‐kidneys was not due to changed protein expression of Erk1/2. In cGKI‐KO‐kidneys, the Erk phosphorylation was not reduced by BAY (Fig. 6B,C).

### Increased IL‐6 levels in cGKI‐KO‐mice

**Figure 7:**
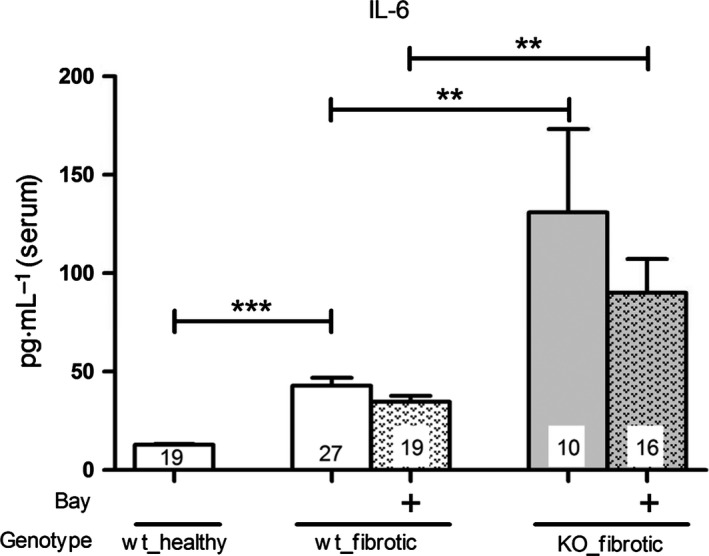
Effect of BAY on the IL‐6 levels in the serum of wt‐ and cGKI‐KO‐mice. The IL‐6 levels in serum of both treated and untreated cGKI‐KO‐mice were significantly higher than in corresponding wt‐mice. However, BAY itself revealed no significant effects in wt‐ and in cGKI‐KO‐mice. Significant differences between two groups are indicated with asterisks (**P < 0.01, ***P < 0.001). The columns show the number of animals which were used. The right columns illustrate the data of GKI‐KO mice and patterned columns the data of BAY‐treated mice.

It has been shown that IL‐6 promotes fibrosis 9. UUO significantly increased the IL‐6 concentration in serum of wt‐mice. Administration of BAY tends to result in diminished IL‐6 levels in serum of wt‐mice compared with untreated wt‐mice. Interestingly, the IL‐6 concentration was significantly higher in untreated and treated cGKI‐KO‐mice and fluctuated much more than in wt‐mice (Fig. 7).

### Effect of BAY and role of cGKI on the renal function examining serum creatinine

**Figure 8:**
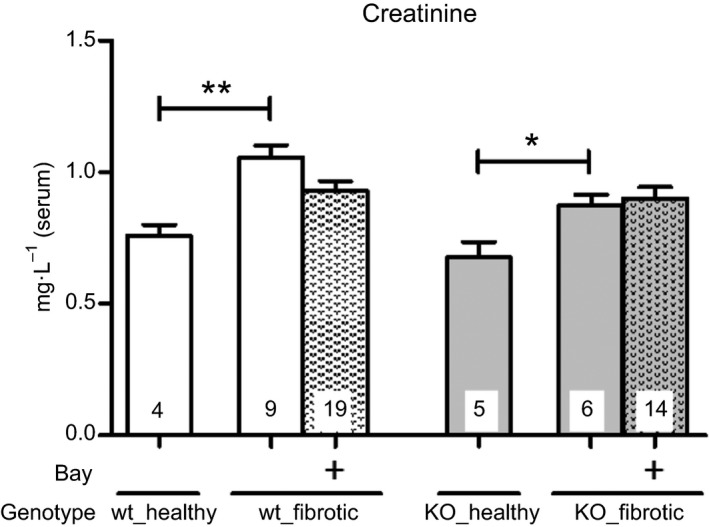
Effect of BAY on serum creatinine in wt‐ and cGKI‐KO‐mice. UUO significantly increased creatinine in the serum of wt‐ and cGKI‐KO‐mice. BAY did not significantly influence creatinine in wt‐mice. Significant differences between two groups are indicated with asterisks (*P < 0.05, **P < 0.01). The columns show the number of animals which were used. The right columns illustrate the data of GKI‐KO‐mice and patterned columns the data of BAY‐treated mice.

The serum level of creatinine increased significantly after 7 days of UUO. Following BAY administration, serum creatinine was decreased, but there was no significant difference between BAY treated and untreated wt‐mice. Conversely, in cGKI‐KO‐mice, BAY influenced in no way the serum creatinine (Fig. 8).

## Discussion

In the present study, we have investigated the functional role of sGC stimulation in regulating renal fibrosis. BAY reduced the mRNA‐ and protein expression of different fibrosis marker. The antifibrotic impact of sGC stimulation was not observed in cGKI‐KO‐mice, suggesting that cGKI mediates the repair process of renal fibrosis.

Our study confirmed that the serum creatinine, which is a parameter for renal function, is increased after UUO 10. However, it was not significantly reduced by BAY in wt‐mice and unchanged in cGKI‐KO‐mice. Our results are in line with the nephroprotective effects of PDE5 inhibitors which also enhance the cGMP pool 11, 12, 13. cGKI‐KO‐mice have higher IL‐6 levels 14, 15 which exert profibrotic effects 9, 16. Conforming with our present study, the IL‐6 levels were increased by UUO and treated, as well as untreated cGKI‐KO‐mice showed a higher IL‐6 concentration than wt‐mice. However, cGKI‐KO‐kidneys revealed no more pronounced fibrosis compared to wt‐kidneys suggesting that other signalling pathways as IL‐6 are important for induction of renal fibrosis. The application of BAY reduced the IL‐6 concentration, but the difference was not significant. Considering the effects of the MAPK signalling, the phosphorylation of Erk promotes fibrosis 17. In cardiac fibrosis the inhibition of Erk phosphorylation by cGMP has already been discussed 18. Our results confirmed the decrease in phosphorylation of Erk after BAY application. Consistent with our data, Beyer *et al*. 19 have also identified that the stimulation of sGC decreased TGFβ signalling through the inhibition of Erk1/2 phosphorylation. Additionally, we observed that cGMP influenced via cGKI the phosphorylation of Erk because in cGKI‐KO‐mice, the effects of BAY were lower.

It is generally accepted that TGFβ acts by stimulation of its downstream mediator smad2 and smad3. Latest studies report that diminished smad2‐ as well as smad3 phosphorylation results in enhanced renal fibrosis 20, 21, 22. However, it is also recently discussed that phosphorylation of smad2 and smad3 by TGFβ exerts reverse effects in renal fibrosis. Smad2 maybe plays a protective role negatively regulating the smad3 signalling. TGFβ activates smad2 which diminishes TGFβ1/smad3 signalling, including phosphorylation, nuclear translocation and the binding of smad3 to the Col1 promoter, leading to augmented collagen synthesis 23, 24. In our study, phosphorylated smad2 was unaffected by cGMP in renal fibroblasts (data not shown). However, nuclear translocation of P‐smad3 was diminished by cGMP in the presence of TGFβ in wt‐, but not in cGKI‐KO‐fibroblasts. Interestingly cGMP inhibited only the translocation of P‐smad3, but not the phosphorylation of smad3 (Fig. S2). In contrast to our study, Beyer *et al*. 19 showed that nuclear P‐smad2‐ and P‐smad3 levels and smad reporter activity were unaffected by sGC stimulation in human fibroblasts. As already mentioned in pulmonary artery smooth muscle cells, activation of cGMP/PKG limited TGFβ‐induced nuclear translocation of smad3 by sequestering smad3 with cytosolic β2‐tubulin 8. However, in contrast we did not detect an increase in P‐smad3–β2‐tubulin interaction after pretreatment with cGMP. In our study exists a cGKIα–P‐smad3–β2‐tubulin interaction in fibroblasts, but the intensity of this interaction is not influenced by cGMP. Consequently, the observed inhibition by cGMP of TGFβ‐induced nuclear translocation of P‐smad3 cannot be explained by sequestering P‐smad3 with cytosolic β2‐tubulin. CTGF is downstream of TGFβ signalling and upregulated in response to TGFβ stimulation 25. However, the regulation of CTGF expression via cGMP is controversially discussed. Hewitson *et al*. and Beyer *et al*. showed that cGMP is not able to decrease the CTGF expression in fibroblasts, 1, 19 which contrasts our study illustrating reduced CTGF expression with BAY.

Expression of PAI‐1, which acts profibrotic, is slightly attenuated by BAY. TGFβ1 activates PAI‐1 and PAI‐1, in turn, stimulates TGFβ1 26. The expression of PAI‐1 is regulated via TGFβ1‐induced Erk phosphorylation 27 which is significantly reduced by BAY.

After UUO, the MMP2 mRNA is adjusted much higher than MMP9. Therefore, MMP2 appears to be more crucial in the development of renal fibrosis than MMP9. Of importance is the fact that BAY‐induced increase in TIMP‐1 expression was accompanied by diminished MMP2 activity. TIMPs do not reveal a high specificity for any particular MMP 28, but we suppose that the diminished activity of MMP2 by BAY maybe caused by the regulation of substantial increased TIMP‐1. The role of MMPs in developing renal fibrosis is very complex and subsequently differently discussed. On the one hand, MMP exert antifibrotic effects degrading diverse components of the ECM. On the other hand, they are implicated in pathological processes such as fibrosis and thereby degrading basal membrane. Especially, MMP2 degrades collagen IV, which is an essential part of the basal membrane 29. A TGFβ‐induced increase of the MMP2 protein and mRNA is also reported 30. In turn, enhanced MMP activity can stimulate the TGFβ‐complex, which afterwards activates fibroblasts and provokes the synthesis of collagen 31. Accordingly, the BAY‐induced reduction of MMP activity may lead to reduced TGFβ activity, which correlates with the observed decreased expression of TGFβ target genes. Considering this, the decrease in MMP2 activity by cGMP/cGKI can ameliorate the progression of renal fibrosis.

BAY application in rats was previously shown to ameliorate renal injury after relief of ureteral obstruction 32. In the clinics, renal damage depends on the duration until relief of ureteral obstruction 33. It has to be evaluated clinically in the future whether application of sGC stimulators might be a therapeutical approach to diminish renal fibrosis upon ureteral obstruction and to enhance renal recovery after relief.

## Conclusion

**Figure 9:**
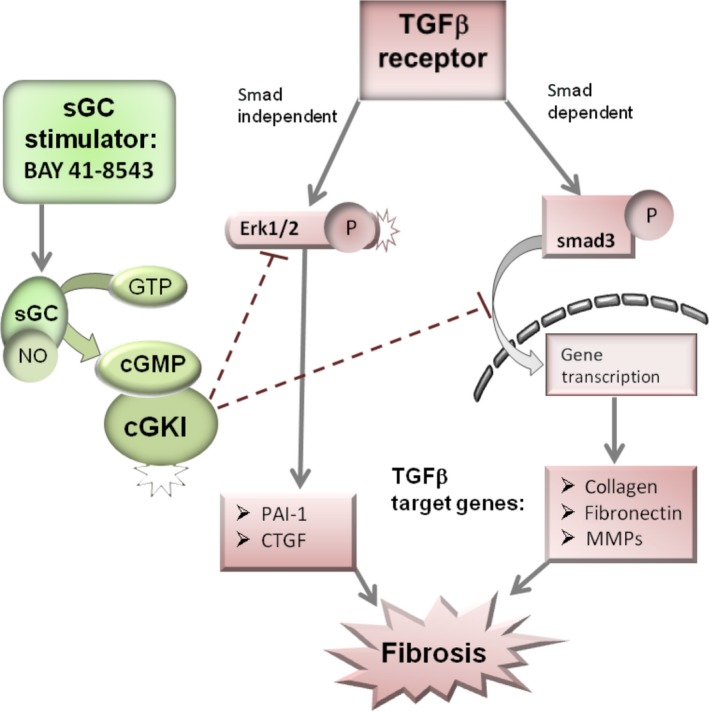
Proposed model demonstrating a possible mechanism for BAY to intervene in the TGFβ signalling pathway in renal fibrosis. Stimulation of sGC by BAY can activate cGKI, which can restrict TGFβ signalling by inhibition of a smad‐dependent pathway to augment target gene transcription, or a smad‐independent pathway which is mediated via Erk1/2. GTP, guanosine triphosphate.

The results of the present study suggest a therapeutic potential of BAY application in renal fibrosis. The antifibrotic effect of BAY is mediated via cGMP/cGKI by inhibition of Erk and smad3 signalling pathways (Fig. 9).

## Materials and methods

### Mice

129/Sv‐WT and 129/Sv‐cGKI‐KO‐mice 34 were bred and maintained in the animal facilities of the University of Regensburg.

The investigation conforms to the guide for the Care and Use of Laboratory Animals published by the US National Institute of Health. The experimental protocols were approved by the local authorities for animal research (Regierung der Oberpfalz, Bayern, Germany; #54‐2532.1‐02/10) and were conducted according to the German law for animal care.

### Unilateral ureter obstruction

The renal fibrosis was induced by UUO in 6–12‐week‐old mice as described in 5. The application with BAY 41‐8543 (BAY; daily, intraperitoneal, 4 mg·kg bw^−1^) started 24 h after surgery using water, glycerol, PEG400 as vehicle. After 7 days, the fibrotic process was analysed.

### Quantitative RT‐PCR

Real‐time PCR of αSMA, fibronectin, Col1a1, CTGF, PAI‐1, TIMP‐1, MMP2, and MMP9 was performed as previously described 5. 18S rRNA served as housekeeper gene. The results are shown as the x‐fold change in mRNA expression ($2^{\Delta \Delta C_{t}}$) in the fibrotic kidney relating to the opposite healthy kidneys whose mRNA expression was set to one.

### Sirius red/fast green

Collagen levels were determined by Sirius red/fast green method 5. We calculated the increase (%) of collagen (collagen/nonprotein collagen) after 7 days UUO related to the healthy kidney.

### Immunofluorescence

The fixation, staining and quantification of kidney tissues were performed as previously reported 5. The quantification of αSMA, fibronectin, Col1a1 and total collagen was related to the contralateral healthy kidney. The fluorescence intensity was quantified using the metamorphic offline software (Visitron Systems, Puchheim, Germany).

### Western blot analysis

The protein expression of CTGF, PAI‐1 (Santa Cruz Biotechnology, Heidelberg, Germany), Erk1/2, P‐Erk1/2 (Cell Signaling, Danvers, MA, USA) and TIMP‐1 (Sigma Aldrich, Taufkirchen, Germany) was assayed by western blotting 5. Representative immunoblots show the influence of UUO in comparison to the contralateral healthy kidney and the effects of BAY in healthy and fibrotic wt‐kidneys. The graphs statistically compare exclusive fibrotic kidneys. The values of all markers of the fibrotic wt‐ and cGKI‐KO‐kidneys were related to the mean values of fibrotic untreated wt‐kidneys. This ratio was set to one and normalized to the corresponding glyceraldehyde‐3‐phosphate dehydrogenase (GAPDH; Cell Signaling) respectively to Erk1/2 for P‐Erk1/2 values. For quantification, ImageJ densitometry was used (BioRad, München, Germany).

### Gelatin zymography assay

The activity of MMP2 and MMP9 was detected using gelatin zymography, which can distinguish between latent and active forms of proteinases.

Briefly, the culture medium was electrophoresed in a SDS/PAGE gel containing 0.1% gelatin. The gel was loaded with 70 μg/35 μg total protein for MMP2/MMP9. The gel was washed (100 mm NaCl and 2.5% Triton X‐100 in 50 mm Tris‐HCl, pH 7.5) to remove SDS and incubated in a reaction buffer (200 mm NaCl, 0.02% NaN_3_, 0.5 μm ZnCl_2_, 1 mm CaCl_2_, 2% Triton X‐100, in 50 mm Tris‐HCl, pH 7.5) for enzymatic reaction at 37 °C overnight. Finally, the gel was stained with Coomassie blue, and destained in 10% acetic acid/30% methanol and quantified using imagej software (open source). MMPs in fibrotic tissue were expressed as relative values of markers in kidneys from untreated wt‐mice.

### Cell culture

The fibroblasts of wt‐ and cGKI‐KO‐mice were isolated and stained as described previously 5. The cells were pretreated with 8Br‐cGMP (Biolog, Bremen, Germany; 1 mm, 1 h, 37 °C) or vehicle followed by exposure to TGFβ (Biomol, Hamburg, Germany; 2 ng·mL^−1^, 1 h, 37 °C) or vehicle. Nuclei were stained with DAPI (gift from Armin Kurtz, University Regensburg). P‐smad3 and P‐smad2 (Cell Signalling), respectively, were detected using an Alexa647‐conjugated anti‐rabbit secondary‐antibody (1 : 200; Invitrogen, Karlsruhe, Germany) for 2 h at room temperature. Coverslips were washed, mounted with glycerol and analysed using an Axiovert 200 microscope (Zeiss, Jena, Germany). To ensure a valid comparison, images were randomly selected from different fields. The intranuclear and extranuclear fluorescence‐intensity of three to six equal areas was measured respectively. Then, the mean value of intranuclear and extranuclear fluorescence intensity of P‐smad3 was determined respectively. For the quantification of the fluorescence intensity all values were related to values of untreated wt‐fibroblasts (control) using the metamorphic offline software.

### (Co‐)immunoprecipitation

The stimulated (TGFβ/cGMP+TGFβ) cells were lysed in 2% Lubrol‐PX buffer [20 mm Tris; 150 mm NaCl, 2% Lubrol (nonaethylenglycol‐monododecylether)] containing phosphatase inhibitors (Roche, Mannheim, Germany) and protease inhibitors. After homogenization and centrifugation (18 000 ***g***, 10 min, 4 °C) the protein concentration of the supernatant was determined by a Lowry‐based method.

The reactions were completed with Co‐IP buffer (50 mm Tris‐HCl, pH 7.5, 15 mm EGTA, 100 mm NaCl, 0.1% Triton X‐100) containing also phosphatase inhibitors (Roche) and protease inhibitors.

About 1000–1500 μg of cell lysates was given onto the beads. Two microgram β2‐tubulin‐antibody (Sigma Aldrich) was added and incubated on ice 90 min. Meanwhile 40 μL of protein‐A‐G‐Sepharose beads (Thermo Scientific, Dreieich, Germany) were pretreated for each immunoprecipitation. They were washed three times with Co‐IP buffer, then blocked with 3% BSA in Co‐IP buffer and washed three times at least once more. After that, the incubated cell lysates were centrifugated (18 000 ***g***, 10 min, 4 °C), then the supernatant was added to the washed and blocked Sepharose beads and rotated overnight at 4 °C. Following this, three washing steps were performed (100 ***g***, 4 °C, 1 min) and the precipitate was eluted with Laemmli buffer 2×. Proteins were separated by SDS/PAGE (12.5%) and blotted to polyvinylidene difluoride membrane (Merck Millipore, Darmstadt, Germany). The blots were incubated with anti‐smad3, anti‐P‐smad3 (Cell Signalling), anti‐cGKIα 35 and anti‐β2‐tubulin, at 4 °C overnight. Bands were visualized by use of an ECL select Western Blotting Detection Reagent (GE Healthcare, Amersham, UK). Coimmunprecipitation of cell extracts without antibody and Co‐IP buffer with antibody, respectively, were used as controls for the specifity of the Co‐IP analysis (data not shown).

### Determination of IL‐6 and cGMP

For measurement of IL‐6 levels in serum, blood was drawn in anaesthetized (2% isoflurane) mice from the retrobulbar plexus and centrifugated (8 min, 1000 ***g***). Afterwards, the IL‐6 levels in the serum were determined with mouse IL‐6 Quantikine ELISA Kit, (R&D Systems, Wiesbaden‐Nordenstadt, Germany). For determination of cGMP concentration in tissue, the kidneys were removed and assessed with cGMP‐EIA kit (IBL, Cayman, UK).

### Serum creatinine

Serum creatinine was determined by HPLC as previously reported with minor modifications 5. In brief, 10 μL serum was mixed with 50 μL perchloric acid to precipitate proteins. The tube was vortexed and kept at 4 °C for 15 min. Following centrifugation (5 min, 10 000 ***g***), an aliquot of 5 μL of the supernatant was injected into the HPLC apparatus (Prominence LC20 series equipped with a LC20A photometric detector set at 234 nm; Shimadzu, Duisburg, Germany). Separation was performed using a Zorbax 300‐SCX 5 μm, 150 × 4.6 mm, analytical column (Agilent, Waldbronn, Germany) and a mobile phase consisting of 5 mm sodium acetate (pH = 5.1)/acetonitrile [800 : 200 (v : v)]. Creatinine eluted after 6.3–6.5 min at a flow rate of 1.0 mL·min^−1^ (column temperature 35 °C).

### Statistical analysis

All data are expressed as mean ± SEM. For calculation of statistical differences between two means, the unpaired Student's *t*‐test (two‐tailed, confidence interval 95%) was used. If the difference between two groups was statistically significant, then it is indicated by asterisks (*P < 0.05; **P < 0.01; ***P < 0.001). *n* indicates the number of animals.

## Conflicts of interest

PS and J‐PS are employees at Bayer Pharma AG. VW is an employee at Novartis Pharma GmbH, Nuremberg. The PhD thesis of VW is funded by Novartis Pharma.

## Author contributions

ES, VW and JS planned experiments, analysed data and wrote the manuscript; ES, VW, AS and FK performed experiments. FH, PS and HPS contributed reagents or other essential material. All authors critically read the manuscript.

## Supporting information


**Fig. S1.** Analysis of renal cGMP levels after BAY application.

**Fig. S2.** Analysis of the whole fluorescence intensity of P‐smad3.
