# Supplementary material for: Inhibition of the TGFβ signalling pathway by cGMP and cGMP‐dependent kinase I in renal fibrosis
Source: FEBS Open Bio. 2017 Mar 1;7(4):550–61. doi: 10.1002/2211-5463.12202 (PMC5377407; doi:10.1002/2211-5463.12202)
Supplement: Supplementary file 1 — Fig. S1. Analysis of renal cGMP levels after BAY application. [file FEB4-7-550-s001.pdf]

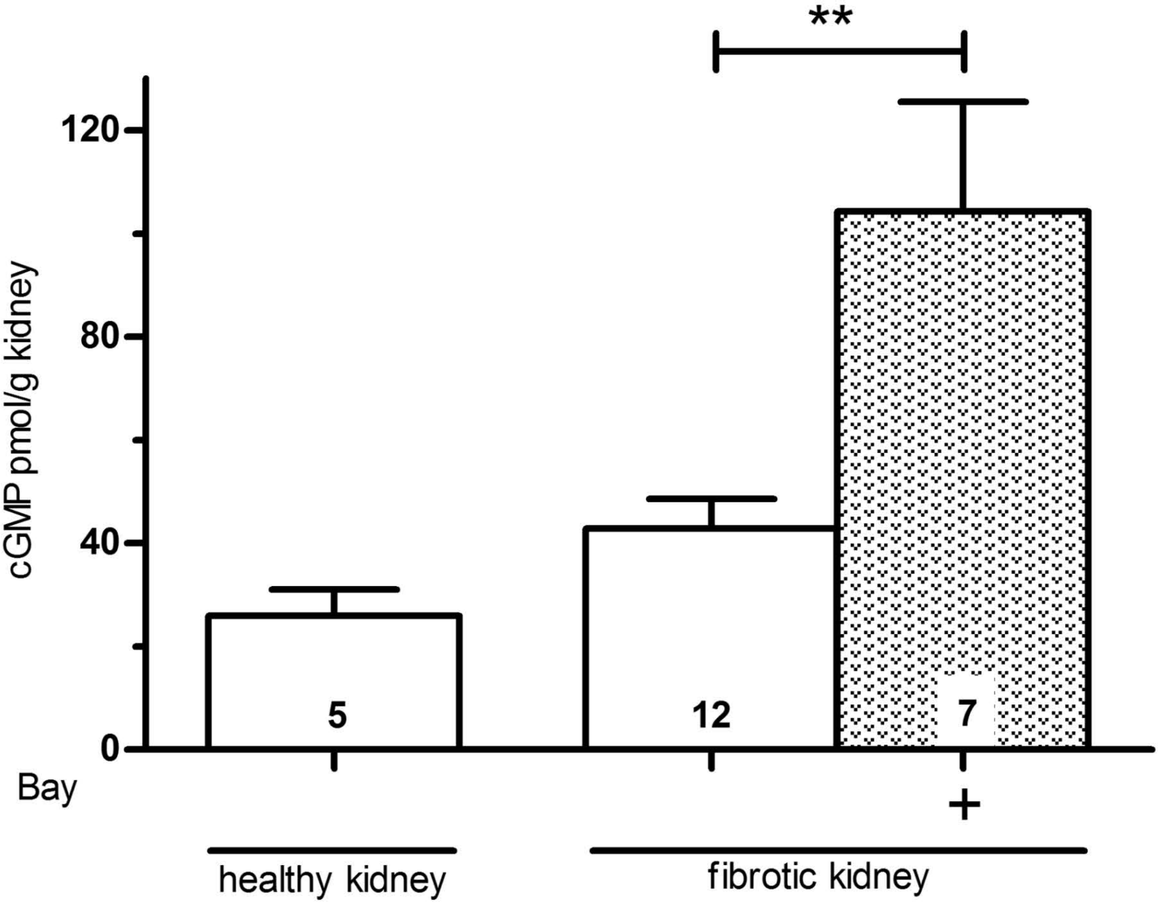

S. Fig. 1

Analysis of renal cGMP levels after BAY application.

BAY application significantly increased cGMP concentration in fibrotic kidney tissues. If the difference between two groups are statistically significant, then it is indicated with asterisks (**$p<0.01$). The columns show the number of animals which were used. The right columns illustrate the data of fibrotic kidneys. The results of BAY treated mice are designated as patterned columns.
