# Supplementary material for: Inhibition of the TGFβ signalling pathway by cGMP and cGMP‐dependent kinase I in renal fibrosis
Source: FEBS Open Bio. 2017 Mar 1;7(4):550–61. doi: 10.1002/2211-5463.12202 (PMC5377407; doi:10.1002/2211-5463.12202)
Supplement: Supplementary file 2 — Fig. S2. Analysis of the whole fluorescence intensity of P‐smad3. [file FEB4-7-550-s002.pdf]

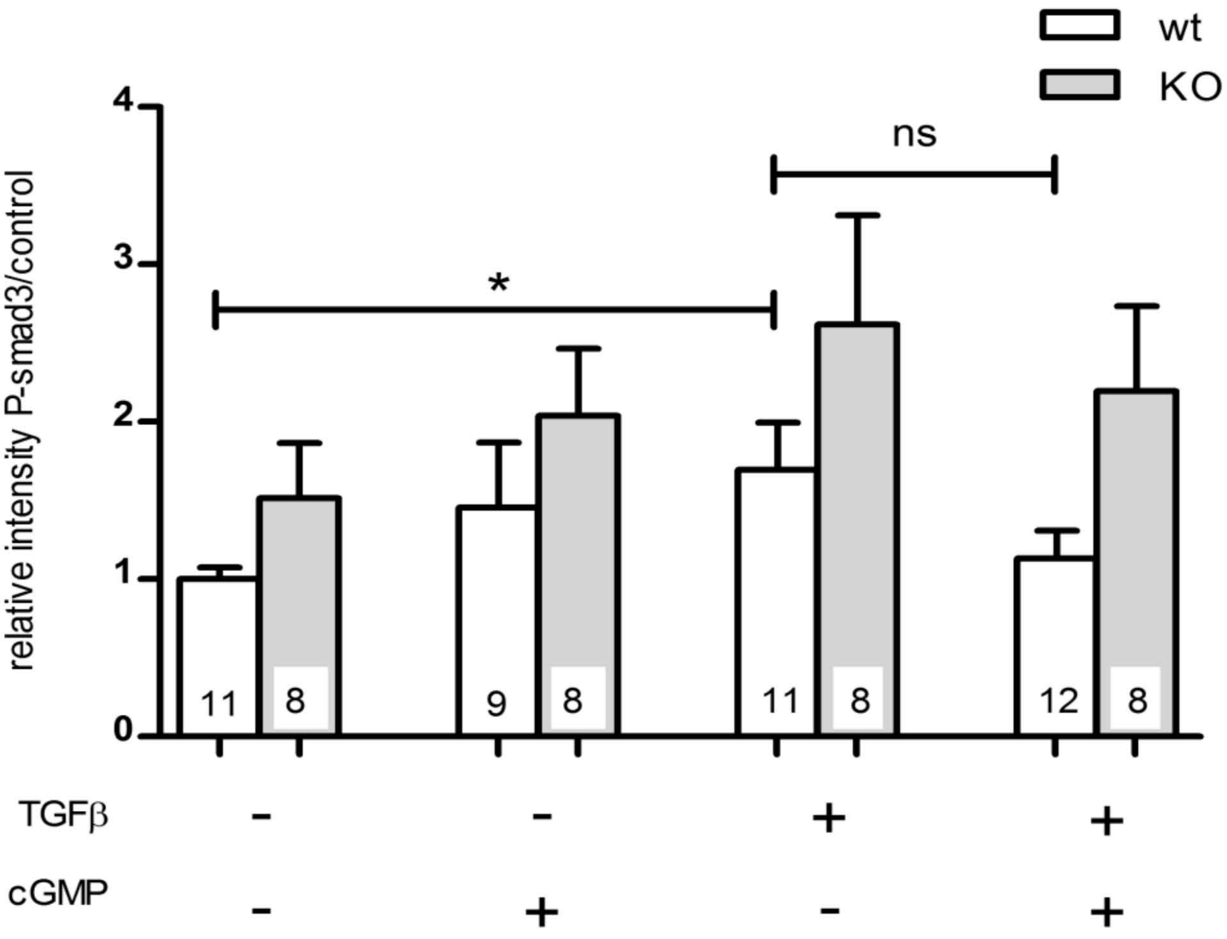

S. Fig. 2

Analysis of the whole fluorescence intensity of P-smad3

The quantification of the relative fluorescence intensity of P-smad3/control is shown in wt- and cGKI-KO-fibroblasts with vehicle, cGMP, TGFβ and cGMP/TGFβ. As control unstimulated wt-fibroblasts were applied. If the difference between two groups is statistically significant, then it is indicated with asterisks (*$p<0.05$). The columns show the number of images which were analysed.
